# ‘RE:fine drugs’: an interactive dashboard to access drug repurposing opportunities

**DOI:** 10.1093/database/baw083

**Published:** 2016-05-17

**Authors:** Soheil Moosavinasab, Jeremy Patterson, Robert Strouse, Majid Rastegar-Mojarad, Kelly Regan, Philip R. O. Payne, Yungui Huang, Simon M. Lin

**Affiliations:** ^1^Research Information Solutions and Innovation, The Research Institute at Nationwide Children's Hospital Columbus, OH 43205, USA; ^2^Division of Biomedical Statistics & Informatics, Mayo Clinic, Rochester, MN 55905, USA; ^3^Department of Biomedical Informatics, The Ohio State University College of Medicine, Columbus, OH 43210, USA

## Abstract

The process of discovering new drugs has been extremely costly and slow in the last decades despite enormous investment in pharmaceutical research. Drug repurposing enables researchers to speed up the process of discovering other conditions that existing drugs can effectively treat, with low cost and fast FDA approval. Here, we introduce ‘RE:fine Drugs’, a freely available interactive website for integrated search and discovery of drug repurposing candidates from GWAS and PheWAS repurposing datasets constructed using previously reported methods in Nature Biotechnology. ‘RE:fine Drugs’ demonstrates the possibilities to identify and prioritize novelty of candidates for drug repurposing based on the theory of transitive Drug–Gene–Disease triads. This public website provides a starting point for research, industry, clinical and regulatory communities to accelerate the investigation and validation of new therapeutic use of old drugs.

**Database URL:** http://drug-repurposing.nationwidechildrens.org

## Introduction

Drug discovery and development is typically a 7–12 year process that requires investment of billions of dollars and abundant clinical trials but results in unpredictable return on investment (1, 2). Drug repurposing—also known as drug repositioning, re-profiling, re-tasking or therapeutic switching (3, 4)—is the process of discovering new indications for already approved drugs to speed up and to lower the cost and risk of developing drugs. Since repurposing relies on previously approved studies that already passed multiple toxicity and other tests, new discoveries tend to be ready for clinical trials quickly and be reviewed by Food and Drug Administration (FDA) faster (4). Approximately 30% of the newly FDA-approved drugs and vaccines in the US in recent years are repurposing discoveries (5). As an example, Pfizer's sildenafil (Viagra) was repositioned from angina treatment to erectile dysfunction treatment in men in 1998 (6).

The widespread practice of drug repurposing has produced a range of computational methods for the discovery of new uses for old drugs. Depending on the availability of the biological and pharmaceutical information, these methods can be drug, disease or target oriented and can be classified into target-based, knowledge-based, signature-based, network-based and targeted-mechanism-based approaches (5, 7). A number of publicly available and searchable drug repurposing databases such as DrugMap Central (8), DRAR-CPI (9), e-Drug3D (10), PharmDB (11) and PROMISCUOUS (12) has proven to be popular and valuable among researchers.

A study by Nelson et al. shows that the proportion of drugs developed with genetic evidence is significantly increasing (13). Recently, Genome-Wide Association Study (GWAS) and Phenome-Wide Association Study (PheWAS) have emerged as target-based methods that have the potential to discover additional disease indications of the same drug target by leveraging the associations between a single gene and multiple diseases (14, 15). In a previous study, we provided a public PheWAS dataset of 52 966 drug–disease candidates for drug repurposing (14). However, that dataset was presented as a static Excel file and it does not include the GWAS dataset.

In this article, we introduce ‘RE:fine Drugs’, an interactive user interface to integrate GWAS and PheWAS reposition datasets using Drug–Gene–Disease triads along with advanced search and export capabilities. Our website enables researchers to explore dataset of drug repurposing pairs to discover novel opportunities for possible treatment of new indications. The proposed usage of ‘RE:fine Drugs’ is under a hypothesis generation framework rather than a statistical testing framework: a positive identification from this database only suggests a possibility of drug reposition, rather than any statistical validation.

## Database creation

**Figure 1.**
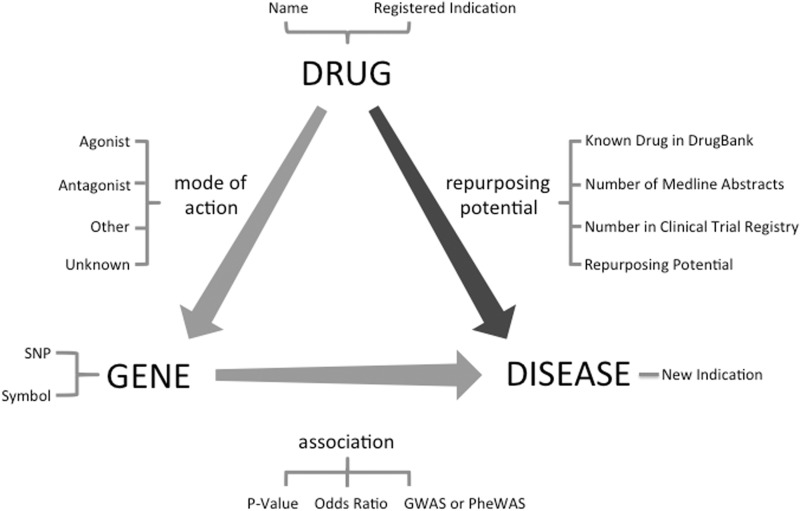
Drug repurposing by the transitive triad of drug, gene and disease. Through the target gene of the drug, new treatment indications can be suggested for an existing drug. This figure shows fields available in ‘RE:fine Drugs’ advanced search.

Identification of candidate drug–disease relationships is the primary step in the drug repurposing task. Although there have been previous studies using Machine Learning-based methods (16) and literature analysis (17) for drug repurposing, we extracted and prioritized a new dataset of candidate drug repurposing pairs using previously established data integration methods (3, 14, 15) using Transitive Property of Equality between drug–gene and gene–disease pairs (Figure 1). The drug–gene relationships were extracted from DrugBank (18), whereas the gene–disease relationships were extracted from GWAS (NIH catalog of 2015) and PheWAS (19) data sources. We first extracted all 212 851 SNP-disease associations from a PheWAS reported by Denny et al. (19) which includes all associations with nominal *P*-value < 0.05. Currently, the PheWAS data is limited to primarily adult patient population from five institutions in the Electronic Medical Records and Genomics (eMERGE) network with a mean age of 69.5 years (19). Afterward, removing any association with no mapping between the SNP and a gene in the dbSNP left us with 48 488 unique gene–disease relationships. Then we used a list of 14 169 direct and indirect gene targets for drugs from DrugBank (18) to identify 52 966 drug–disease relationships. We followed a similar approach for GWASs. We retrieved all gene–disease pairs from GWAS catalog and using drug–gene target pairs from DrugBank, we identified 7945 potential drug–disease pairs.

Under the hypothesis-generation framework, we chose a loose *P*-value cutoff of 0.05 (without adjusting multiple testing) to create a list of gene–disease associations, and then propagate the claim through the drug–gene–disease triad. Thus, we test the effectiveness of our framework to enrich drug reposition candidates using a permutation test. We use the co-occurrence of a drug and a disease in biomedical literature and clinical trials database as a proxy of biological plausibility of the drug reposition, since it can be influenced by literature bias (20, 21). Our study shows that 28% (14 816 out of 52 966) of the transitive drug–disease pairs from PheWAS were supported in at least one Medline abstract while permuting drug–disease pairs will only identify 6.2% of random drug–disease words in the literature. We applied the same method using data extracted from GWAS catalog and verified co-occurrence of 38.8% (3087 out of 7945) drug–disease pairs in Medline abstracts while 6.2% co-occurrence identified using shuffled drug–disease pairs. These findings suggest that PheWAS and GWAS data can significantly enrich drug–disease pairs to be further considered for the potential of reposition.

Furthermore, to estimate the novelty of the drug–disease pairs and to prioritize them, we cross referenced all pairs with the NIH clinical trial registry (i) and also used the biomedical literature co-occurrences of drug–disease pairs (ii) to categorize each PheWAS and GWAS generated pair in one of these four categories: ‘known/rediscovered’ for relationships that already exist in the DrugBank, ‘strongly supported’ for pairs with some evidence in both (i) and (ii), ‘Likely’ for pair with support in either (i) or (ii) and ‘novel’ for pairs with no evidence in (i) or (ii). Note that the lack of occurrence of both drug and disease terms together in the literature could potentially indicate a novel finding, a bias of literature annotations or a true lack of association (20, 21).

## Design and implementation

With regard to the design of ‘RE:fine Drugs’, we sought to provide a friendly user interface for the researchers to interactively filter drug repurposing candidates and browse them in a prioritized fashion. The auto-complete feature allows the users to search for drugs, diseases and genes by prefixes. In addition, users can search for candidates with strong support in the literature or clinical trials database, or restrict the results to the ones with significant *P*-value scores. All the search and suggestion functionalities are implemented with Edge-N-Grams and multiple inverted indexes using Elasticsearch (https://www.elastic.co/products/elasticsearch) and the user interface and backend are benefiting from the non-blocking and asynchronous concepts of node.js (https://www.nodejs.org) to offer a fast and fresh navigation to the users.

### Case study

**Figure 2.**
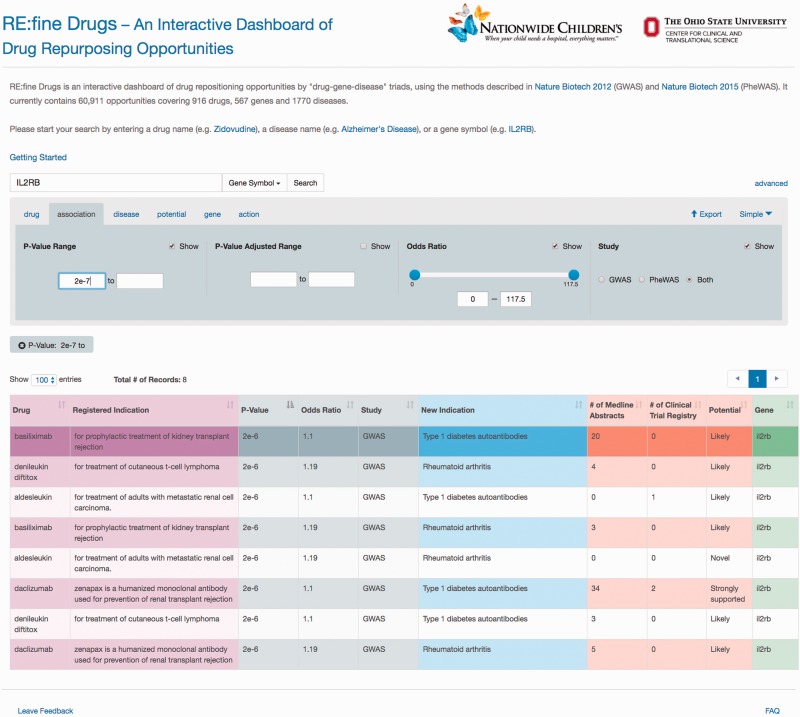
A sample screen shot of the advanced search options and the result table for gene ‘IL2RB’ and *P*-value greater than 2e−7. To find and prioritize the repurposing opportunities, users can toggle between the advanced search tabs to change the search criteria and use the pagination and sorting options to browse the results.

‘RE:fine Drugs’ promises delivery of prioritized drug–disease candidates for drug repurposing. For example, a user will find 12 pairs of drug–disease associations by searching for the ‘IL2RB’ gene. The user can then use the advanced search and results table to filter and sort results by *P*-value, odds ratio, literature support, clinical trials, disease name and/or drug name. Several results for the IL2R monoclonal antibody ‘daclizumab’ are returned, which was originally developed for the prevention of organ transplant rejection. One example of an interesting drug repurposing hypothesis for daclizumab that has been validated in the literature is its use in treating asthma via acting as an immunosuppressant (22–24). A sample screen shot of this search scenario and the results is shown in Figure 2.

Furthermore, the search system accommodates different entry points using drugs, genes or diseases, which enable a multidisciplinary research team of chemists, molecular biologists, pathologists and other researchers to extract drug repurposing candidates, find the association information and export the results for further analysis.

Please note that the *P*-values in Figure 2 are nominal *P*-values reported from the SNP-disease association studies from either GWAS or PheWAS; they do not directly test the association between drug and repurposed disease indication. To generate hypothesis for drug repurposing candidates, many researchers prefer to use nominal *P*-values to protect from type II errors of missing an opportunity (25). However, there are also concerns of finding spurious associations due to multiple testing (26). As such, we also provide false-discovery rate (FDR) adjusted *P*-values under the advanced options tab. The selection of a drug repurposing candidate for further clinical test can be largely a business decision, for instances, cost, market size and competitors. As such, we reemphasis that the reported *P*-values are only for a guidance of hypothesis generation rather than hypothesis testing.

## Future work

This study is sought to provide an easy and fast access to the databases of drug repurposing potentials and enables researchers to explore and share these opportunities. We aimed to balance the timeliness of the system availability and richness of capabilities. There are a couple of enhancements we are planning for already.

The result table in the current design contains the number of co-occurrences of drug and disease terms in Medline and clinical trial registry. However, these results do not provide the user with a link to the Medline abstract or clinical trials registry. In the future, we plan to provide list of links to these resources along with a short snippet of text so that researchers can investigate the validity and directionality of the drug–disease co-occurrence evidence and causality of the SNP/gene in question.

In our current design, researchers can sort the results by couple of categories such as the ‘New Use’, but they are limited to sort disease names alphabetically. In the next release, we intend to integrate a disease ontology framework so that users can search and sort by categories such as ‘cancer’, ‘neurological disease’, etc. and we automatically expand the categories to include all the diseases in that category.

As more researchers start to use the system and provide feedbacks, we can address additional enhancements as needed.
